# Screening of Bacteria Promoting Carbon Fixation in *Chlorella vulgaris* Under High Concentration CO_2_ Stress

**DOI:** 10.3390/biology14020157

**Published:** 2025-02-03

**Authors:** Chuntan Chen, Yu Wang, Qunwei Dai, Weiqi Du, Yulian Zhao, Qianxi Song

**Affiliations:** 1 School of Environment and Resource, Southwest University of Science and Technology, Mianyang 621002, China; tansic@foxmail.com (C.C.); 19808119360@163.com (Y.W.); dwq_0811@163.com (W.D.); zhaoyuhao123you@163.com (Y.Z.); westqianxi@163.com (Q.S.); 2 Key Laboratory of Low-Cost Rural Environmental Treatment Technology at Sichuan University of Arts and Science, Education Department of Sichuan Province, Sichuan University of Arts and Science, Dazhou 635000, China; 3 Key Laboratory of Exploitation and Study of Distinctive Plants in Education Department of Sichuan Province, Sichuan University of Arts and Science, Dazhou 635000, China; 4 School of Foreign Languages and Cultures, Southwest University of Science and Technology, Mianyang 621002, China

**Keywords:** *Chlorella vulgaris*, CO_2_ fixation, high CO_2_ concentration, microalgae-bacterial system, symbiotic bacteria

## Abstract

The cooperation between microalgae and bacteria can enhance the carbon fixation efficiency of microalgae. In this study, a microalgae-bacteria coexistence system under high-concentration CO_2_ stress was constructed, and the bacterial community structure of the entire system was analyzed using the 16S rDNA technique. *Microbacterium* sp., *Bacillus* sp., and *Aeromonas* sp. were screened and demonstrated to promote carbon fixation in *Chlorella vulgaris* HL 01 (*C. vulgaris* HL 01). Among them, the *Aeromonas* sp. + *C. vulgaris* HL 01 experimental group exhibited the most significant effect, with an increase of about 24% in the final biomass yield and a daily carbon fixation efficiency increase of about 245% (day 7) compared to the control group. Continuous cultivation of microalgae and bacterial symbiosis showed that bacteria could utilize the compounds secreted by microalgae for growth and could produce nutrients to maintain the vitality of microalgae. Detection of extracellular organic compounds of microorganisms in the culture broth by excitation-emission matrix spectral analysis revealed that bacteria utilized the aromatic proteinaceous compounds and others secreted by *C. vulgaris* HL 01 and produced new extracellular organic compounds required by *C. vulgaris* HL 01. The metabolic organic substances in the liquids of the experimental groups and the control group were analyzed by liquid chromatography-mass spectrometry, and it was found that 31 unique organic substances of *C. vulgaris* HL 01 were utilized by bacteria, and 136 new organic substances were produced. These differential compounds were mainly organic acids and their derivatives, benzene compounds, and organic heterocyclic compounds, etc. These results fully demonstrate that the carbon fixation ability and persistence of *C. vulgaris* HL 01 are improved through material exchange between microalgae and bacteria. This study establishes a method to screen carbon-fixing symbiotic bacteria and verifies that microalgae and bacteria can significantly improve the carbon fixation efficiency of microalgae for high-concentration CO_2_ through material exchange, providing a foundation for further research of microalgae-bacterial carbon fixation.

## Simple Summary

Microalgae have been employed to fix high-concentration CO_2_ in order to lower industrial CO_2_ emissions. However, the majority of research concentrates on the fixation of CO_2_ by microalgae, neglecting the role that bacteria play in this process. In this study, the microalgae-bacterial community system underwent stress acclimation using a high concentration of CO_2_. Three bacterial strains that can significantly enhance the CO_2_ fixation of *Chlorella vulgaris* were effectively screened out after thorough investigation and analysis. On this basis, techniques such as excitation-emission matrix spectroscopy and liquid chromatography-mass spectrometry were used to confirm the exchange of various organic compounds between microalgae and bacteria during carbon fixation. This material exchange mechanism effectively enhances the carbon fixation ability of *Chlorella vulgaris*. This study offers novel approaches and research ideas for microalgae carbon fixation, which should encourage more advancement and application in this field.

## 1. Introduction

As is well known, microalgae can convert CO_2_ into biomass through photosynthesis. As global temperatures continue to rise due to significant CO_2_ emissions, the fixation of high-concentration CO_2_ by microalgae in industrial applications has become a hot research topic [1]. Previous studies on CO_2_ fixation by microalgae have typically considered only the single biological role of microalgae. Various studies have sought to improve carbon fixation efficiency through the optimization of parameters [1], mutagenesis [2], and device modification [3], with some limited progress. However, these studies were limited to analyzing the microalgae in isolation without considering the potential effects of bacteria on the growth and CO_2_ fixation of microalgae in the carbon fixation system. Some studies even ensured the exclusion of bacterial contributions by sterilizing the culture solution before experiments [1,4]. Most projects using microalgae to fix high-concentration CO_2_ use open systems such as raceway ponds [5]. Various types of bacteria can be symbiotic with microalgae. Even the relatively closed column and plate photobioreactor systems inevitably have bacteria in the culture system in outdoor engineering applications [6]. These bacteria can affect CO_2_ fixation by microalgae [7], but the details of their effects remain poorly understood.

Microalgae-bacterial symbiosis exists in natural or artificial systems for treating wastewater, and there is growing interest in increasing the efficiency of microalgae-bacterial symbiosis for improved wastewater treatment [8]. Attempts to improve the efficiency of wastewater treatment have characterized the flora and screened bacteria [9]. Microalgae-bacterial consortium systems have been shown to have better removal efficiency for some typical contaminants of pollutants in municipal, industrial, and agricultural wastewater [10,11]. Researchers have also studied these systems to explore the underlying mechanisms of microalgae-bacterial interactions and to screen bacteria by resolving the structure of inter-microalgae flora [12]. For example, an analysis of the inter-microalgae flora of wastewater treatment systems identified the main microbial populations that act on sulfonamides in wastewater [13]. In another study, an analysis of the microbial community structure of *Chlorella* culture was used to isolate bacteria that promote the growth of microalgae [14].

The pressure of global warming has prompted some researchers to explore CO_2_ fixation by microalgae-bacterial associations, but most studies have focused on the removal of pollutants from water [15]. Using industrial wastewater as the culture medium, a co-culture of native bacteria with *Spongiochloris* sp. not only removed organic pollutants, but also achieved CO_2_ fixation [16]. *Chlorella* sp. and *Scenedesmus obliquus* were co-cultured with bacteria isolated from activated sludge to effectively treat pig wastewater, removing 63.48% of CO_2_ [17]. Sepehri et al. used *Chlorella vulgaris* and activated sludge isolates for CO_2_ fixation while treating municipal wastewater [18]. These studies provide strong evidence that microalgae-bacterial systems can achieve CO_2_ reduction and suggest new strategies to utilize CO_2_ fixation by microalgae. Unfortunately, all of these studies performed CO_2_ fixation using microalgae with mixed bacterial populations and did not isolate single bacteria that could promote CO_2_ fixation by microalgae after adaptive culture of microalgae-bacterial symbiosis at the high CO_2_ concentrations typical of industrial applications.

In the microalgae-bacterial symbiosis system, microalgae and bacteria engage in extensive material exchange [19]. The dissolved organic carbon and proteins produced by microalgae through photosynthesis are secreted extracellularly, thereby providing essential nutrients for symbiotic bacteria. Upon utilizing these substances, bacteria produce micronutrients and vitamins [20,21], such as vitamin B_12_, plant hormones (indole-3-acetic acid, abscisic acid, cytokinin, ethylene, and gibberellin), thiamine derivatives, and siderophores, which in turn foster algal growth [22]. Studies have revealed that soil rhizobia are capable of producing B_12_, which is utilized by freshwater green microalgae (*Lobosomonas rostrata*) for their growth. Meanwhile, bacteria rely on organic matter extracted from microalgae as their carbon source [23]. However, the research on the material exchange between different algae and bacteria is not detailed enough, particularly regarding the material exchange under high-concentration CO_2_ stress.

In view of the potential of the microalgae-bacterial symbiotic system in the field of CO_2_ fixation, screening symbiotic bacteria that can significantly enhance the carbon-fixation efficiency of microalgae and exploring the interaction mechanisms between them are of great significance for both academic research and practical applications. This study established an artificial microalgae-bacterial symbiosis system and acclimated *Chlorella vulgaris (C. vulgaris)* and bacteria under high CO_2_ concentration for long-term growth. Through analyzing the bacterial community associated with the algae, three strains of bacteria were identified that promote the fixation of high-concentration CO_2_ by *C. vulgaris*. By examining the effectiveness of co-culturing *C. vulgaris* and bacteria in fixing 15% (*v*/*v*) CO_2_, we explored the material exchange between them, thereby providing a research foundation for enhancing carbon fixation in microalgae-bacteria systems.

## 2. Materials and Methods

### 2.1. Strain and Culture Medium

The microalgae strain used in this study was *Chlorella vulgaris* HL 01 (*C. vulgaris* HL 01), isolated from bicarbonate-rich natural waters in western China to better adapt to high bicarbonate content in the culture medium. *C. vulgaris* HL 01 can thrive in a culture medium abundantly supplied with HCO_3_^−^ (>15 mmol L^−1^) [24]. 

Selenite Enrichment medium (SE medium, a carbon-free culture medium [25]) was used as a carbon fixation medium for *C. vulgaris* HL 01. A gas mixture of 15% (*v*/*v*) CO_2_ (approximately the concentration typical of exhaust flue gas from coal-fired power plants [2]) was the only carbon source and was composed of pure CO_2_ mixed with air sterilized by ultraviolet radiation.

### 2.2. Stress Culture of C. vulgaris and Bacteria at a High CO_2_ Concentration

A 150 cm high, 20 cm diameter column photobioreactor (open top) was used, containing 40 L of sterilized SE medium. *C. vulgaris* HL01 and water from a municipal wastewater treatment plant sedimentation tank were added. The reactor was equipped with a microporous aeration disk at the bottom to bubble 15% (*v*/*v*) CO_2_ at a flow rate of 0.3 L L^−1^ min^−1^. The culture was incubated under controlled natural light (with the natural light irradiation controlled in the range of 0–12,000 lux), temperature (15–27 °C), and relative humidity (40–80%) for 120 days under natural light (light-dark ratio approximately 12–14 h:10–12 h) for *C. vulgaris* HL 01 domestication. Microalgae and bacteria were cultured under high concentration CO_2_ stress for 120 days.

### 2.3. Isolation and Screening of Symbiotic Bacteria

The mixture of microalgae and bacteria after long-term cultivation was diluted in a ten-fold gradient. The diluted solution was spread on LB medium agar plates (peptone 10.0 g L^−1^, yeast extract 5.0 g L^−1^, NaCl 10.0 g L^−1^) and incubated in the dark at 30 °C for 3 days in a constant temperature incubator (MI-150, SHENGYUAN, Zhengzhou, China) until single colonies appeared. Single colonies were purified using the streak plate method and inoculated into liquid LB medium for further experiments. The specific method can be referred to in the literature [26].

### 2.4. Analysis of Bacterial Strain Growth Characteristics

The bacterial culture was inoculated into LB liquid culture medium, and grown with shaking for 28 h at 30 °C before Gram staining and observation under a fluorescence microscope (MicroPublisher 5.0 RTV, Olympus, Shinjuku City, Japan). To measure growth, 0.4 mL of freshly inoculated bacterial broth was transferred to individual wells of a 96-well bacterial culture plate, and the plate was placed in a fully automated growth curve analyzer (FP-1100-C, BIOSCREEN, Turku, Finland), with optical density values (*DO_600_*) determined every 30 s.

### 2.5. Microbial Diversity Analysis

Total genomic DNA was extracted from samples using the CTAB method. The extraction process was as follows. The sample was placed in a 2 mL centrifuge tube, and 1 mL of CTAB extraction buffer (2%) and lysozyme (5 μL, 50 mg L^−1^) were added, and then the mixture was incubated in a water bath at 65 °C for 1 h, with inversion and mixing every 15 min. After centrifugation, the sample (1 mL) was then mixed with phenol (pH 8.0): chloroform: isoamyl alcohol (25:24:1), followed by centrifugation at 12,000 rpm for 10 min. The sample-containing layer of the supernatant was then mixed with chloroform: isoamyl alcohol (24:1), and centrifuged at 12,000 rpm for 10 min. The supernatant containing the sample was transferred into a new 1.5 mL centrifuge tube, and then an equal volume of isopropanol was added. Subsequently, the mixture was shaken and incubated at −20 °C for 20 min. After centrifugation at 12,000 rpm for 10 min, the liquid was carefully poured off, taking care not to lose the precipitated material. The pellet was then washed twice with 1 mL of 75% ethanol, followed by centrifugation and aspiration of the remaining liquid. The DNA sample was dissolved in double-distilled water before sequencing, with incubation for 10 min at 55–60 °C to aid dissolution. The purified material was then sent to Beijing Novogene Technology Co., Ltd (Building 301, Yard 10A, North Jiuxianqiao Road, Chaoyang District, Beijing, China). for sequencing and analysis.

The V4 hypervariable region of the bacterial 16S rRNA gene was amplified using the specific primers 515F (5′-GTTTCGGTGCCAGCMGCCGCGGTAA-3′) and 806R (5′-GCCAATGGACTACHVGGGTWTCTAAT-3′). The PCR products were analyzed by 2% agarose gel electrophoresis, and qualified PCR products were further purified using magnetic beads and quantified by enzyme-linked immunosorbent assay. Prior to loading onto a 2% agarose gel, the purified samples were thoroughly mixed in equal amounts (determined based on the concentration of the PCR products). The PCR products were detected by agarose gel electrophoresis, and the target bands were recovered using a gel extraction kit. The TruSeq^®^ DNA PCR-Free Sample Preparation Kit was used for library construction. The constructed libraries were quantified using Qubit and real-time quantitative PCR. After quantification, the libraries were sequenced on the NovaSeq 6000 sequencer (Illumina, San Diego, CA, USA). The sequencing data were analyzed using the Novogene Magic platform. The specific method can be referred to in the literature [27].

### 2.6. 16S rDNA Sequence Analysis and Construction of Phylogenetic Tree

DNA of isolated bacteria was extracted using AxyPrep Bacterial Genomic DNA Miniprep Kits (AXYGEN, Glendale, AZ, USA) according to the manufacturer’s protocol. The 16S rDNA was PCR-amplified with the primers 27F (5′-AGAGTTTGATCMTGGCTCAG-3′) and 1492R (5′-TACGGYTACCTTGTTACGACTT-3′). After the amplified products were purified with AxyPrep DNA Gel Extraction Kits (AXYGEN, USA), the DNA fragments were sequenced by Beijing Novogene Technology Co., Ltd. (Beijing, China). All sequences were compared to the NCBI 16S rRNA sequences database using the basic local alignment search tool (BLAST). A phylogenetic tree was constructed using the neighbor-joining method in MEGA 7.0.26 [28]. Bootstrap resampling analysis was used to estimate the confidence of tree topologies (1000 replicates).

### 2.7. C. vulgaris-Symbiotic Bacteria CO_2_ Fixation Experiment

To assay fixation, 60 mL of log-phase *C. vulgaris* HL 01 (*OD_680_* = 0.1) was mixed with 3 L of SE medium [29]. Subsequently, 12 mL of different bacteria (*OD_600_* = 0.1) were added to each experimental group respectively (12 mL of sterilized LB medium was added to the control group), and the samples were incubated in an artificial climate chamber (BIC-400, Shanghai Boxun Industry & Commerce Co., Ltd. Medical Equipment Factory, Shanghai, China) using 15% (*v*/*v*) CO_2_ as the carbon source. The incubation conditions were an inlet flow rate of 0.1 L L^−1^ min^−1^, a temperature of 25 °C, a light intensity of 5000 lux, and a light:dark ratio of approximately 12 h:12 h.

### 2.8. Measurement and Calculation of CO_2_ Fixation by Microalgae-Bacterial Symbiosis System

100 mL of the mixed microalgae solution was taken every two days, centrifuged at 1000 rpm for 15 min, and then the supernatant was discarded. The sediment was washed three times with deionized water and dried at 70 °C in a constant temperature drying oven (LC-202-00B, LICHEN, Changsha, China). The dried sample was weighed with an electronic scale (HC-625e, Hochoice, Shanghai, China) and the dry weight was recorded once it had dried to a consistent weight.

The fixation efficiency of *C. vulgaris* HL 01 was calculated using the following equation [30]:

$$R_{{CO}_{2}}=C_{avg}\times \frac{{DWB}_{2}-{DWB}_{1}}{t_{2}-t_{1}}\times \frac{{MW}_{{CO}_{2}}}{{MW}_{C}}$$

where, *DWB* is the dry weight of biomass, mg L^−1^; *t*_1_ and *t*_2_ are the starting and ending points, respectively. *R*_*CO*_2__ is CO_2_ fixation, mg L^−1^ d^−1^; *C_avg_* is the average content of elemental carbon in microalgae biomass measured by an elemental analyzer, set at 50% [25,31]. *MW*_*CO*_2__ is the molecular weight of CO_2_. *MW_C_* is the molecular weight of representative carbon.

### 2.9. Excitation–Emission Matrix Spectral (EEM) Analysis

For excitation–emission matrix spectral (EEM) analysis, 10 mL of microalgae culture solution was centrifuged at 6000 rpm for 5 min, and the resulting supernatants were used for measurement in a fluorescence spectrophotometer (F7000, HITACHI, Chiyoda City, Japan). Fluorescence intensity was measured at excitation wavelengths from 200 nm to 600 nm in increments of 5 nm and at emission wavelengths from 200 nm to 600 nm in increments of 5 nm. Before spectral analysis, any negative values in the data were set to zero.

### 2.10. Analysis of Metabolic Organics in Culture Medium

After 13 days of culture, the medium was centrifuged at 1000 rpm for 10 min. After removing the sediment, the supernatant was centrifuged again at 2000 rpm. A 10 mL aliquot of the supernatant was collected and frozen at −80 °C. The frozen samples were sent to Shanghai Majorbio Technology Co., Ltd (Lane 3399, Kangxin Highway, International Medical Park, Pudong New Area, Shanghai, China). for analysis of metabolic compounds using UHPLC-Q Exactive HF-X (Thermo Fisher Scientific, Waltham, MA, USA). The specific steps are as follows: chromatographic conditions: 3 μL of sample was separated on a HSS T3 column (100 mm × 2.1 mm i.d., 1.8 µm; Waters, Milford, MA, USA) and then subjected to mass spectrometry detection. Mobile phase A consisted of 95% water and 5% acetonitrile (containing 0.1% formic acid), while the mobile phase B was made up of 47.5% acetonitrile, 47.5% isopropanol, and 5% water (containing 0.1% formic acid). The flow rate was maintained at 0.40 mL min^−1^, and the column temperature was set at 40 °C. Mass spectrometry conditions: The mass spectrum signal was collected using positive and negative ion scanning modes, with a mass scanning range of 70–1050 *m*/*z*. The sheath gas flow rate is maintained at 50 psi, while the auxiliary gas flow rate stands at 13 psi. The auxiliary gas heating temperature is set at 425 °C. The ion spray voltage for the positive mode is adjusted to 3500 V, while for the negative mode, it is set to −3500 V. Additionally, the ion transfer tube temperature is maintained at 325 °C, and the normalized collision energy is set to a cyclic pattern of 20–40–60 V. The resolution of the primary mass spectrometry is 60,000, while that of the secondary mass spectrometry is 7500. Data were collected using data-dependent acquisition (DDA) mode. The liquid chromatography-mass spectrometry (LC-MS) raw data underwent multiple processing steps using the metabolomics processing software Progenesis QI (Waters Corporation, Milford, MA, USA).

### 2.11. Statistical Analysis

The data analysis of microbial diversity and metabolomics was carried out using R 4.3.3 software. The visualization of microbial diversity was conducted using Canoco 5.0 software, while the remaining figures were drawn using Origin 2021. Each experiment was repeated three times, and all statistical results were expressed in the form of mean ± standard deviation.

## 3. Results and Discussion

### 3.1. Structure of Artificially Established Integral Microbial Communities

**Figure 1:**
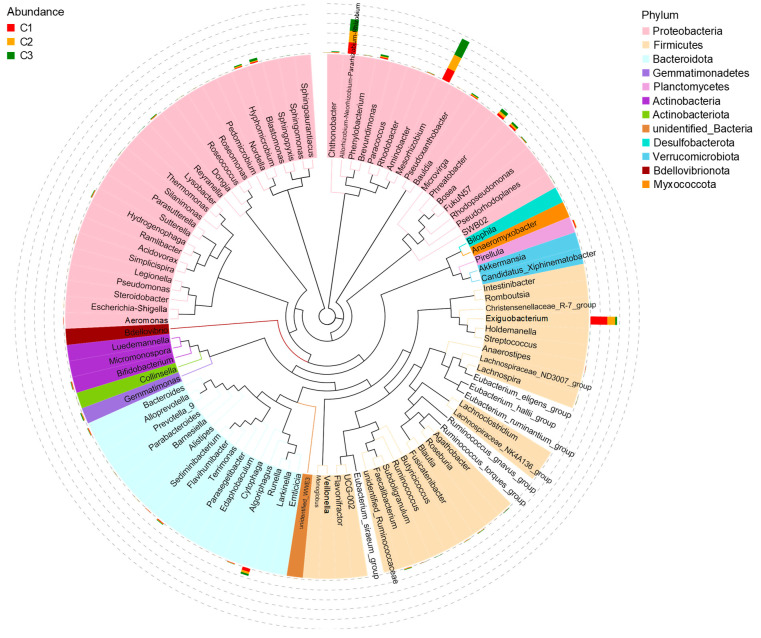
Evolutionary relationships at the genus level in the artificially established microalgae-bacterial system: The phylogenetic tree was constructed from representative sequences at the genus level, with the colors of branches and sectors indicating their corresponding phyla. The stacked bar charts on the outer rim of the sectors represent the abundance distribution of the genera across different samples.

To obtain a relatively stable artificial microalgae-bacterial system, three parallel systems, C1, C2, and C3, were established under the same experimental conditions. The community structures of the final cultured microalgae solutions were analyzed. In the three parallel groups, there were several bacterial phyla identified. Bacterial phyla with relative abundances greater than 1% included *Proteobacteria*, *Firmicutes*, and *Bacteroidota*. Among them, about half were *Proteobacteria*, and the other two phyla had relatively low abundance ratios (Figure 1).

In this experiment, municipal wastewater plant sedimentation tank effluent was used as the starting inoculum. Therefore, it is not surprising that the relative abundances of *Proteobacteria*, *Firmicutes*, and *Bacteroidota* were relatively high, as these are the common dominant phyla used with *Chlorella* for wastewater treatment [32]. Phyla with relative abundances less than 1% in the system include *Planctomycetes* [33], *Actinobacteria* [32], *Gemmatimonadetes*, *Actinobacteriota*, *Desulfobacterota* [34], *Verrucomicrobiota* [35], *Bdellovibrionota* and *Myxococcota* [36], which are common in microalgae systems. These bacteria were able to survive in *C. vulgaris* cultures with 15% (*v*/*v*) CO_2_ as the only carbon source, indicating a symbiotic or commensal relationship with *C. vulgaris* [37].

To understand the distribution of specific genera, the microorganisms in the experimental groups were further analyzed. As shown in Figure 1, *Mesorhizobium* accounted for the largest proportion of bacteria in all three experimental groups. Recent studies have reported that this genus can promote nitrogen fixation and growth in a variety of green microalgae and co-culturing with *Mesorhizobium* sp. increased *C. vulgaris* biomass by 66.3% and oil content by 47.7% [33]. The *Allorhizobium*-*Neorhizobium*-*Pararhizobium*-*Rhizobium* clade was also present at a high percentage, and these bacteria were previously shown to promote the growth of microalgae [14]. *Phreatobacter* was also identified here and was previously detected in the presence of microalgae [13]. *Bose*, *Brevundimona*, *Blastomonas*, and *Hyphomicrobium* were also detected.

Figure 1 shows that among the *Firmicutes*, the *Exiguobacterium* that can grow in the same system as *C. vulgaris* HL 01 is the most dominant species [38]. For instance, co-cultivating *Exiguobacterium aurantiacum* with *Chlorella* could increase the amount of chlorophyll a + b produced by the latter [39]. In addition, previous research has shown that *Runella* in *Bacteroidota* can also increase the CO_2_ fixation capacity of microalgae [40]. Overall, analysis of the culture subjected to high CO_2_ concentration revealed that the microorganisms in the established artificial microalgae-bacterial system evolved toward a population of bacteria favorable to *C. vulgaris* growth and CO_2_ fixation. This can facilitate further screening of symbiotic bacteria with probiotic effects on *C. vulgaris*.

### 3.2. Characterization of Bacteria That Promote Carbon Fixation in C. vulgaris HL 01

**Figure 2:**
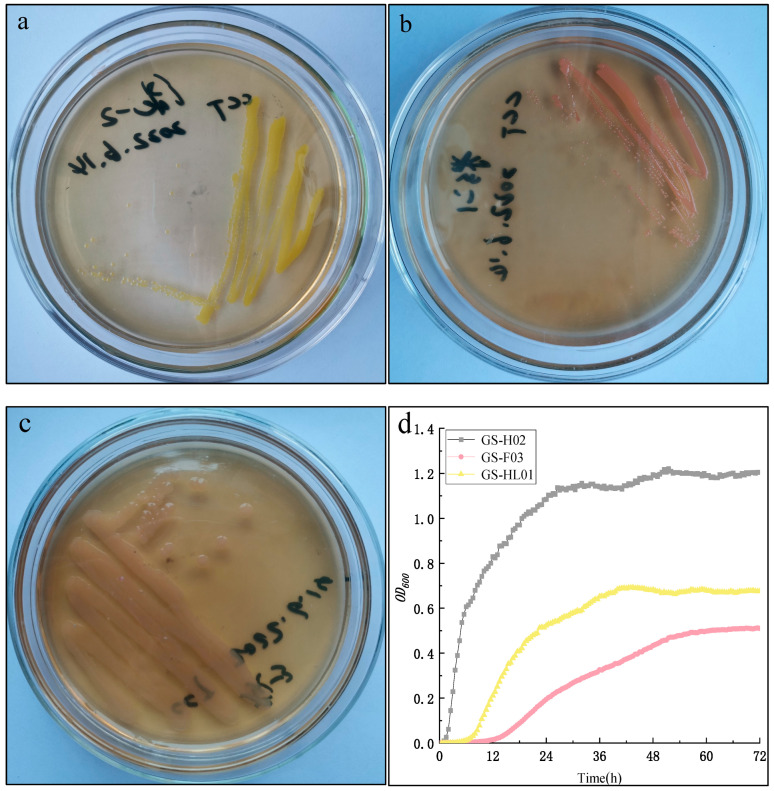
Growth on solid media and growth curves in liquid media of three bacterial strains: (**a**) GS-H02, (**b**) GS-F03, (**c**) GS-HL01, (**d**) growth curve.

After 120 days of acclimation, multiple strains of bacteria symbiotic with *C. vulgaris* HL 01 were successfully isolated from the artificial microalgae-bacteria system. Then, three strains with the best carbon fixation effect were selected and numbered as GS-H02, GS-F03, and GS-HL01. Through microscopic observation, all three isolated strains were short rod-shaped bacteria. Figure 2a shows that GS-H02 is a yellow, Gram-positive bacterium; Figure 2b shows that GS-F03 is pink; and Figure 2c shows that GS-HL01 is a gray, Gram-negative bacterium.

A comparison of the growth curves of the three strains of bacteria (Figure 2d) reveals that GS-H02 had the shortest lag phase, lasting 0–2 h; GS-HL01 exhibited a moderate lag phase, lasting 0–8 h; and GS-F03 had the longest lag phase, lasting 0–14 h. Through the stress domestication in this experiment, the logarithmic growth periods of the three strains were relatively long, with GS-H02 lasting approximately 24 h during the period of 2–26 h, GS-HL01 lasting 32 h during the period of 8–40 h, and GS-F03 lasting up to 38 h during the period of 14–52 h. After entering the stable stage, the three strains of bacteria remained active until 72 h without any decay phenomenon. Thus, the three isolates have long logarithmic and stable periods, which differs from what was seen for bacteria isolated in previous studies with only a few hours of growth period [41]. The durations of the logarithmic and stable periods of the three strains of bacteria are all above 24 h and 20 h, respectively, resulting in a significant extension of the growth cycle. This result may be due to the lack of environmental nutrients in the closed system. The bacteria could only use the organic materials released by *C. vulgaris* as a carbon source because organic carbon was not provided to the cultivation system. *C. vulgaris* produced relatively little organic matter in the early stages of culture. To adapt to the growth cycle of *C. vulgaris*, the three strains gradually acclimate to genetic changes to adapt to a longer nutrition supply cycle as a way to extend the overall growth cycle.

### 3.3. 16S rDNA Identification and Phylogenetic Tree

**Figure 3:**
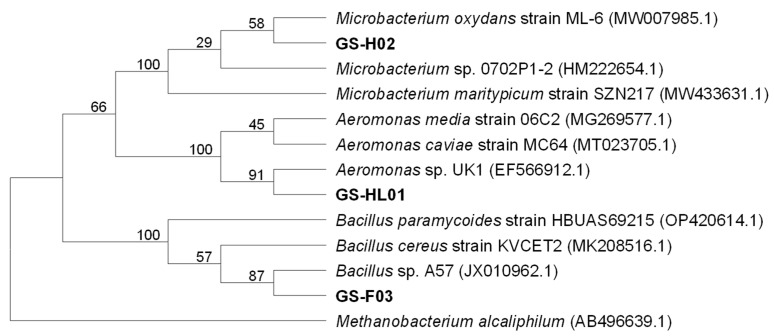
Phylogenetic tree of 16S rRNA gene sequences of three bacterial strains: The isolated strains are shown in bold.

Phylogenetic tree analysis (Figure 3) showed that GS-H02 belonged to *Microbacterium* sp., and it was closely related to *Microbacterium oxydans* (58%), GS-HL01 was closely related to *Aeromonas* sp. (91%), and GS-F03 was closely related to *Bacillus* sp. (87%). *Microbacterium* sp. and *Aeromonas* sp. are common bacteria found in natural waters. In fact, it has been well-documented that they have been isolated from outdoor cultivations of microalgae [42]. *Microbacterium* sp. can produce indole-3-acetic acid (IAA) to promote *Chlorella* recombinant phosphoglycolate phosphatase activity and contribute to *Chlorella* growth [43]. The results of further metabolic analysis indicate that the *Microbacterium* sp. screened out in this experiment is indeed capable of producing IAA and related substances. Clagnan et al. detected high abundance of *Aeromonas* sp. in the photobioreactors where algae thrives, which proved that *Aeromonas* sp. could co-exist with microalgae in culture medium [44]. Unfortunately, further research on whether *Aeromonas* sp. has a promoting effect on microalgae has not been conducted. In this experiment, *Microbacterium* sp. and *Aeromonas* sp. were co-cultured with *C. vulgaris* in an environment where CO_2_ was the sole inorganic carbon source. The growth of these bacterial strains indicates that these two strains of bacteria can only utilize the organic substances produced by *C. vulgaris* as a carbon source. Moreover, the newly produced substances by the bacteria may, in turn, serve as a nutrient source that promotes the growth of *C. vulgaris*. The strains isolated here were from a mixed environment containing microalgae and bacteria that likely underwent genetic mutations after 15% (*v*/*v*) CO_2_ treatment, potentially altering microbial activities. Future work should include an in-depth comparative study at the genetic level. Previous studies demonstrated that *Bacillus cereus* treated with γ-ray irradiation was used in the cultivation of *Chlorella*, which not only increased the biomass of *Chlorella* but also promoted the production of its lipids [45].

*Bacillus megaterium* is a plant-promoting bacterium that produces IAA and vitamin B_12_, and promotes *Chlorella* growth through substance exchange [46]. Compared to the other two strains, *Bacillus* sp. was previously shown to significantly promote the growth of *C. vulgaris* in co-culture with a dissolved carbon source, consistent with the result in this study.

### 3.4. Fixation of 15% (v/v) CO_2_ by Microalgae-Bacterial Symbiosis

**Figure 4:**
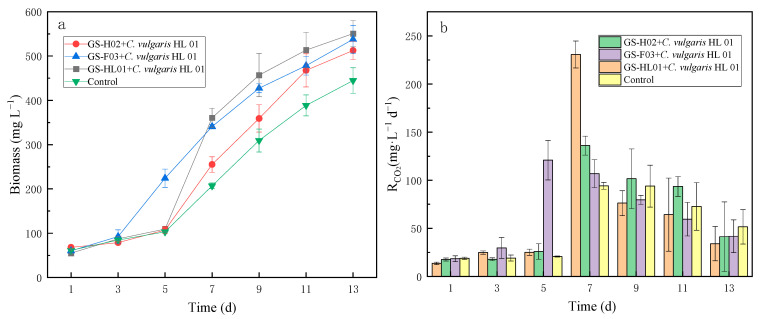
Biomass and CO_2_ fixation rate: (**a**) Biomass of *C. vulgaris* HL 01 and bacteria, (**b**) CO_2_ fixation rate of *C. vulgaris* HL 01.

*C. vulgaris* is composed of lipids, proteins, and polysaccharides, all containing carbon [31], and the main element of bacteria is also carbon. In this experiment, CO_2_ was the sole source of carbon, and the carbon source required for the growth of *C. vulgaris* and bacteria was converted from CO_2_. Biomass was measured as an indicator of the growth of *C. vulgaris* and bacteria. The growth of *C. vulgaris* and bacteria indirectly reflects the efficiency of CO_2_ fixation by *C. vulgaris* [30]. Total biomass was significantly higher in the presence of bacteria compared to the control group without bacteria after 13 days of incubation. The culture with GS-HL01 + *C. vulgaris* HL 01 was approximately 24% higher, while those with GS-F03 + *C. vulgaris* HL 01 and GS-H02 + *C. vulgaris* HL 01 were about 21% and 15% higher, respectively (Figure 4b). The findings show that, in contrast to earlier culture techniques, *C. vulgaris* and bacteria can encourage mutual growth while employing high quantities of CO_2_ as the only carbon source, which is different from the methods used in previous culturing [26]. In the first 3 days, *C. vulgaris* HL 01 did not produce enough organic matter for bacterial growth, so the growth of *C. vulgaris* HL 01 was not affected by bacteria, and there was little difference in biomass between the experimental groups and the control group. As the experiment continued, *C. vulgaris* HL 01 used photosynthesis to convert CO_2_ into organic matter that was secreted as extracellular organic compounds. Bacteria used the extracellular organic compounds as a carbon source and these bacteria also produced material that promoted the growth of *C. vulgaris* HL 01 in microalgae-bacterial symbiosis [47]. The GS-F03 + *C. vulgaris* HL 01 group started to grow rapidly from day 3, so it can be inferred that *Bacillus* sp. more efficiently used the extracellular organic compounds and produced substances to promote *C. vulgaris* HL 01 growth. Faster growth of *C. vulgaris* HL 01 in the GS-HL01 + *C. vulgaris* HL 01 and GS-H02 + *C. vulgaris* HL 01 group started at 5 days, indicating that *Microbacterium* sp. and *Aeromonas* sp. had longer response times to the utilization of extracellular organic compounds of *C. vulgaris* HL 01 and had a certain adaptation period.

The addition of bacteria increased the CO_2_ fixation efficiency of *C. vulgaris* HL 01 (Figure 4b). The fixation rate of CO_2_ was not high for the first 3 days, during which the control group and all experimental groups were in the acclimatization period. At the 5th and 7th days (during the logarithmic growth phase), the experimental groups outperformed the control group in terms of CO_2_ fixation rate. All experimental groups showed a faster decline in CO_2_ fixation by the 9^th^ day when compared to the control group. Thus, the presence of bacteria promoted microalgae CO_2_ fixation in the pre-experimental period. This may be because in the fixed culture system, the rapid growth of *C. vulgaris* quickly consumes the available nutrients, and the growth becomes limited by the scarcity of nutrients. Thus, there is a decreased secretion of extracellular organic compounds, which is required to promote bacterial growth, so the bacteria cannot feed back to promote the fixation of CO_2_ by *C. vulgaris*. The highest single-day CO_2_ fixation rate was achieved by the GS-HL01 + *C. vulgaris* HL 01 group on day 7. Although it did not reach the 1.00 mg L^−1^ d^−1^ CO_2_ fixation rate reported in other studies, it was about 245% higher than the control group. GS-F03 + *C. vulgaris* HL 01, the lowest of the experimental group, was also about 128% higher than the control group. This provides a reference for a further selection of the optimal culture conditions. Combining these three strains with *C. vulgaris* HL 01 to fix 15% (*v*/*v*) CO_2_ each demonstrates that growing bacteria and microalgae together is more effective at allowing microalgae to fix large concentrations of CO_2_.

### 3.5. Analysis of Extracellular Secretions of Microalgae-Bacterial Systems

To further investigate the effect of the three bacterial strains on *C. vulgaris* and to analyze possible interactions between microalgae and bacteria, the experimental groups and control group were separately incubated in BG11 culture for 20 consecutive days. The control group showed yellowing of the *C. vulgaris* HL 01 culture, while the experimental groups grew well although the biomass did not increase. In the control group, *C. vulgaris* HL 01 grew to a late stage where the necessary nutrients were depleted and growth was limited, causing some *C. vulgaris* HL 01 to enter the decay stage, decreasing chlorophyll and resulting in the gradual yellowing of *C. vulgaris* HL 01 [48]. Although the experimental groups were also limited by nutrients, the bacteria could secrete small amounts of nutrients that could be used for *C. vulgaris* growth (e.g., vitamin B_12_ and trace elements). These nutrients were previously shown to maintain the viability of *Chlorella* and prevent *Chlorella* death in acidic environments [21,22].

**Figure 5:**
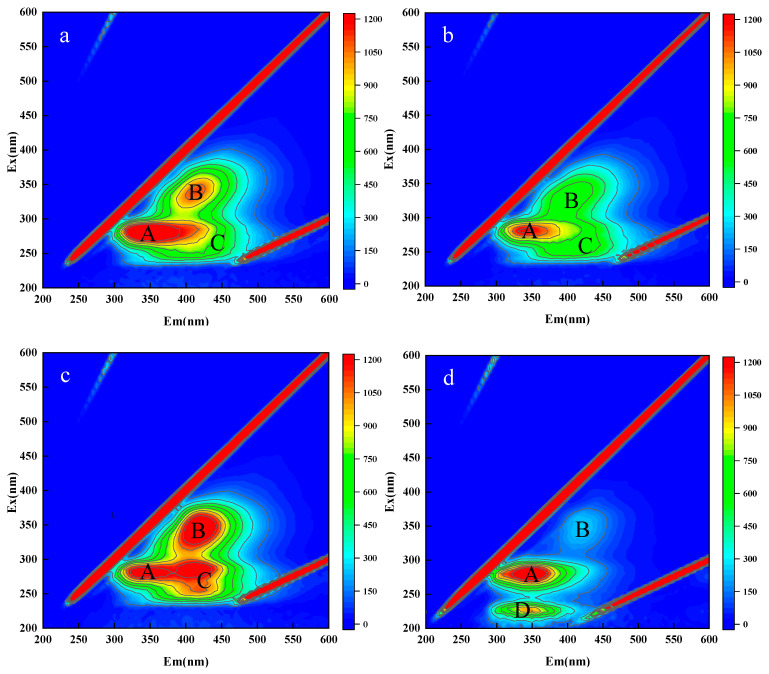
EEM of the microalgae-bacterial system and culture solution of a sterile microalgae system: (**a**) GS-H02 + *C. vulgaris* HL 01, (**b**) GS-F03 + *C. vulgaris* HL 01, (**c**) GS-HL01 + *C. vulgaris* HL 01, (**d**) control.

EEM testing was conducted on the microalgae culture medium supernatant to explore the extracellular products involved in the microalgae-bacteria interaction. The EEM shown in Figure 5 were compared with the standard spectrum. There were significant differences in the extracellular organic compounds of the experimental groups and that of the control group.

The strongest peaks were found in the A region for all samples, with the production of Tyrosine, Protein-like, and Tryptophan-like substances. The secretion of these substances by *C. vulgaris* is consistent with the results of previous studies [49]. Further analysis of the A region showed that the GS-H02 + *C. vulgaris* HL 01 group wave was larger, indicating that *Microbacterium* sp. secreted these organic substances, while the GS-F03 + *C. vulgaris* HL 01 group exhibited the lowest intensity of this region in all samples, indicating that *Aeromonas* sp. consumed these substances [50].

The B region was dominated by Humic acids [51], and the experimental groups had significantly stronger waves than the control group. Although *C. vulgaris* can secrete these substances, this is more likely secreted by bacteria (Figure 5). *Aeromonas* sp. can produce organic substances, such as humic acids, so the peak intensity of the GS-HL01 + *C. vulgaris* HL 01 group in this area is higher. The peaks of the GS-H02 + *C. vulgaris* HL 01 group *(Microbacterium* sp., G^+^) and the GS-F03 + *C. vulgaris* HL 01 group (*Bacillus* sp., G^+^) in region B were significantly lower than those in the GS-HL01 + *C. vulgaris* HL 01 group (*Aeromonas* sp., G^−^), indicating that Gram-negative bacteria were more prone to produce humic acid than Gram-positive bacteria in the symbiotic environment of microalgae and bacteria.

The C region was mainly dominated by bacterially secreted humic acid-like and fulvic acid-like substances, as well as hydrophobic acid, which can promote microalgae growth and prevent the death of *C. vulgaris* [52]. Previous studies found that hydrophobic acids were detected in microalgae culture, which also confirmed the results of this experiment [53]. All experimental groups produced secretions in this area. Since the bacteria in the GS-HL01 + *C. vulgaris* HL 01 group are Gram-negative, their secretion waves are the strongest. Peaks were largely absent in the control group since there was no bacterial involvement.

The control group showed a strong wave in the D region dominated by aromatic protein, while the experimental groups showed no significant wave in this region. Based on the results, it can be inferred that *C. vulgaris* secretes aromatic proteins, which can be utilized as essential organic compounds for bacterial growth [54]. Only high concentrations of macromolecular organic compounds can be detected by EEM representing only one aspect of the microalgae-bacterial interaction, as microalgae-bacteria may secrete trace organic compounds that can promote material exchange and signal transmission. Although these trace organic compounds are typically present at low concentrations, they may contribute to microalgae-bacterial symbiosis and should be investigated in future work.

### 3.6. Differential Analysis of Metabolites in the Culture Medium

**Figure 6:**
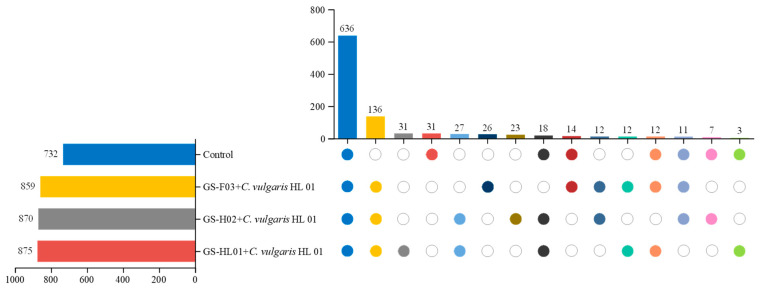
Upset diagram of metabolic compounds in different experimental groups and control group: The left figure shows metabolite counts for the control and experimental groups: the X-axis represents metabolite numbers (bar graphs), and the Y-axis indicates treatment groups. The right figure depicts metabolite intersections between the control and experimental groups: the X-axis denotes intersection types, the upward Y-axis shows intersection counts (bar graphs), and the downward Y-axis corresponds to the treatment groups from the left figure. Circles represent participation in intersection analysis (colored solid circles for participation, hollow circles for non-participation).

Only 732 compounds were found in the microalgae control group, compared to 875 compounds in the GS-HL01 + *C. vulgaris* HL 01 group, 870 compounds in the GS-H02 + *C. vulgaris* HL 01 group, and 859 compounds in the GS-F03 + *C. vulgaris* HL 01 group, according to the differential analysis of metabolites between microalgae and bacteria (Figure 6). All experimental groups and the control group shared 636 substances, primarily amino acids, carboxylic acids, neurotransmitters, fatty acids, vitamins, and so forth.

Despite their ability to generate a variety of amino acids, microalgae have trouble reusing these amino acids [55]. Based on metabolite analysis in the control group, 31 compounds, including organic acids and derivatives, benzenoids, and organoheterocyclic compounds, were found to be missing from all experimental groups. Among these substances, amino acids make up the majority. Likewise, 136 compounds that were absent from the control group were detected in all experimental groups; they primarily included organic acids and derivatives, organoheterocyclic compounds, lipids and lipid-like molecules, benzenoids, organic oxygen compounds, phenylpropanoids and polyketides, and other compounds. As is widely known, bacterial denitrification can transform amino acids into organic acids, alcohols, and lipids [56,57]. We can speculate that the control group has a higher amount of lysine when comparing the types and quantities of compounds in the experimental and control groups. Bacteria may use aspartate, histidine, threonine, valine, and other amino acids to transform them into phospholipids, carboxylic acids, and other substances, and create new nitrogen-containing compounds. These novel nitrogen-containing substances can provide *C. vulgaris* with a nitrogen source for long-term growth once more [58]. We suggest that, in the co-culture system of microalgae and bacteria, the compounds generated by microalgae metabolism may be used by bacteria to create new compounds that microalgae can reuse, even though the growth of microalgae is restricted by the nutrients that are required. These substances have the capacity to keep *C. vulgaris* growing steadily. Bacteria can enhance the way microalgae use organic matter in water, increasing the algal density in the culture system, according to earlier research [59].

Co-cultivating bacteria and microalgae can not only convert nutrients but also generate plant stimulants that encourage microalgae growth. In this study, plant growth enhancers like IAA were discovered in all experimental groups. The control group had tryptophan and indolepyruvate, which generate IAA, but did not have these plant growth stimulators. This shows that bacteria use *C. vulgaris* metabolites to create new chemicals that encourage the growth of microalgae [60]. A comparison of the microalgae growth in the experimental and control groups (Figure 4) reveals that bacteria and microalgae use metabolites in tandem, which encourages microalgae development.

Furthermore, a number of distinct compounds were discovered in several experimental groups, including 31 in the GS-HL01 + *C. vulgaris* HL 01 group, 23 in the GS-H02 + *C. vulgaris* HL 01 group, and 26 in the GS-F03 + *C. vulgaris* HL 01 group. The distinct chemicals found in various experimental groups might be either created by bacteria on their own or secreted by microalgae following the use of the metabolites produced by bacteria. More investigation on this issue is warranted.

## 4. Conclusions

In this study, an artificial microalgae-bacteria co-culture system was established to screen for bacteria that enhance CO_2_ fixation by *C. vulgaris*, and to identify the types and species of compounds that are mutually utilized by microalgae and bacteria. The research shows that a variety of bacteria were found to coexist with microalgae. Among them, the three strains of bacteria screened can grow by utilizing the metabolites of *C. vulgaris*, such as aromatic proteins. Meanwhile, they secreted compounds such as humic acid-like substances, fulvic acid-like substances, aquatic acidic substances, and IAA, which promote the growth of *C. vulgaris* and enhance the utilization efficiency of high-concentration CO_2_. Compared to traditional microalgae-based carbon fixation methods, the synergistic carbon fixation by microalgae and bacteria represents a more promising biological approach. This study has significant potential applications in reducing high-concentration industrial CO_2_ emissions, mitigating global warming, and enhancing the carbon sequestration capacity of microalgae.

## Data Availability

Data in this study are available from the corresponding author upon reasonable request.
